# Pathophysiology and Clinical Impacts of Chronic Kidney Disease on Coronary Artery Calcification

**DOI:** 10.3390/jcdd10050207

**Published:** 2023-05-10

**Authors:** Zhuoming Dai, Xiangyu Zhang

**Affiliations:** Department of Geriatrics, The Second Xiangya Hospital, Central South University, Changsha 410011, China

**Keywords:** vascular calcification, coronary artery calcification, chronic kidney disease, cardiovascular disease, vascular smooth muscle cells

## Abstract

The global prevalence of chronic kidney disease (CKD) has increased in recent years. Adverse cardiovascular events have become the main cause of life-threatening events in patients with CKD, and vascular calcification is a risk factor for cardiovascular disease. Vascular calcification, especially coronary artery calcification, is more prevalent, severe, rapidly progressive, and harmful in patients with CKD. Some features and risk factors are unique to vascular calcification in patients with CKD; the formation of vascular calcification is not only influenced by the phenotypic transformation of vascular smooth muscle cells, but also by electrolyte and endocrine dysfunction, uremic toxin accumulation, and other novel factors. The study on the mechanism of vascular calcification in patients with renal insufficiency can provide a basis and new target for the prevention and treatment of this disease. This review aims to illustrate the impact of CKD on vascular calcification and to discuss the recent research data on the pathogenesis and factors involved in vascular calcification, mainly focusing on coronary artery calcification, in patients with CKD.

## 1. Introduction

Chronic kidney disease (CKD) is a major social and public health issue worldwide. Cardiovascular diseases (CVDs) are some of the serious complications of CKD. Despite recent advances in therapy technologies, the incidence of CVDs continues to increase annually, seriously affecting the health of the population [1]. Vascular calcification is closely associated with the development of CVDs, such as coronary atherosclerosis, myocardial infarction, and malignant arrhythmias. Compared to the general population, vascular calcification is more common and severe in patients with CKD, which is a high-risk factor for cardiovascular complications [2] and greatly increases the incidence of death due to CVD [3]. Cardiovascular-related lesions and adverse cardiovascular events are the main factors that influence the life expectancy of over 50% uremia patients [4].

The most dangerous and fatal cardiovascular events are coronary-artery-related diseases, in which coronary artery calcification (CAC) plays an important role. CAC is formed because of the ectopic deposition of calcium salts in the walls of coronary vessels. Recent studies have shown that CAC formation is not just a passive process of calcium overdeposition, but an active process similar to bone and cartilage formation with multiple factors involved, and the key link is the transformation of vascular smooth muscle cells (VSMCs) to osteoblast-like cells and the continuous expression of related calcifying proteins [5,6]. Calcification can be divided into intimal and medium calcification. Intimal calcification is often closely related to endothelial cell damage and dysfunction and atherosclerosis. Its development is mainly regulated by the inflammatory response, and often distributed in a punctate or lamellar form [7], similar to endochondral ossification, which usually occurs during the lipogenesis phase. Medium calcification, also known as Monckeberg’s-type sclerosis, is more closely linked to CKD, hyperparathyroidism, abnormal calcium and phosphorus metabolism, diabetes mellitus, and osteoporosis, and is also associated with osteogenic-like transformation and the aging of VSMCs [8]. In the past, it was thought that mesenteric calcification might be a benign lesion, but now it has been found that mesenteric lesions can also lead to vascular wall sclerosis and cause adverse cardiovascular events [9].

CAC is closely associated with the development of coronary heart disease (CHD) [10]. CAC can lead to coronary artery wall stiffness, reduce compliance, and cause poor myocardial perfusion and other bad effects; it can effectively reflect the presence and load level of atherosclerotic lesions and validly indicate the risk of adverse cardiovascular events [11,12]. CAC is highly prevalent and dangerous in patients with CKD, and exploring CAC interventions and related therapeutic targets of these patients have become hotspots for research. In patients with CKD, there are some unique CAC risk factors that vary from other underlying diseases. Unlike in the general population, the development of CAC in CKD is largely due to their complex metabolic environment and physiological processes similar to the ossification caused by an imbalance of calcification inhibitory and promotion factors. Further elucidation between the relationship of CKD and CAC will contribute to the management and treatment of CVDs, especially CHD, in patients with CKD. Thus, in this review, we discuss vascular calcification, especially CAC, in patients with different stages of CKD and analyze the possible risk factors and related mechanisms underlying this process.

## 2. Impact of CKD on CAC

CKD is defined as renal damage, with an estimated glomerular filtration rate (eGFR) < 60 mL/min/1.73 m^2^ for a period usually greater than three months [13]. As the kidney function deteriorates, electrolyte disturbances, particularly hyperphosphatemia, hypocalcemia, and metabolic acidosis, gradually appear. Decreased renal excretion, metabolic dysfunction, increased uremic toxins, endocrine dysfunction (e.g., decreased erythropoietin secretion), and decreased expression of inhibitory mineralization factors predispose to renal osteodystrophy. Chronic renal failure (CRF) is a severe form of CKD; this group of patients usually have an active vitamin D deficiency, which can cause secondary hyperparathyroidism and renal osteodystrophy. Malnutrition, microinflammation, water and sodium retention are common in these patients.

The environment in CKD promotes the advancement of vascular calcification, especially CAC. CAC is independently and dramatically associated with the development of CVD in patients with CKD [14]. The all-cause mortality due to coronary artery disease was significantly higher in CKD patients with a higher CAC score (CACS) [15]. Patients with CKD have a higher risk of all-cause mortality and hospitalization for cardiovascular disease when CACS > 400 [16].

Many patients with CKD develop diffuse CAC prior to dialysis. A prospective study that enrolled 117 non-dialysis CKD patients showed that the incidence of CAC was as high as 48%, where 21% had severe CAC (≥400), and a higher CACS was linked to a higher incidence of cardiovascular events and death [17]. In another study, high CACS was found to be a significant predictor of the progression to the renal replacement stage in follow-up CKD patients [18].

CAC affects the progression of renal disease in turn. A lower eGFR was closely related to a higher CACS (≥400), and a higher CAC load was linked to poor prognosis in the CKD population, with an increased high-sensitivity C-reactive protein (CRP) level [19]. According to Yun [20], a high CACS was associated with a significantly higher risk of sustained CKD progression and progressively worsening renal outcomes. Another prospective study reported that CAC plays a strong role as an independent predictor of end-stage renal disease (ESRD) and mortality in patients with CKD stages 3–5. Patients who progressed to ESRD at the fastest rate either had the highest CACS or the most severe CKD-induced mineral and bone disorders (MBDs) [21].

CKD is a condition with the potential for negative feedback in which it induces vascular calcification and in turn worsens the kidney function.

### 2.1. Possible Mechanisms of CKD on Vascular Calcification

**Figure 1:**
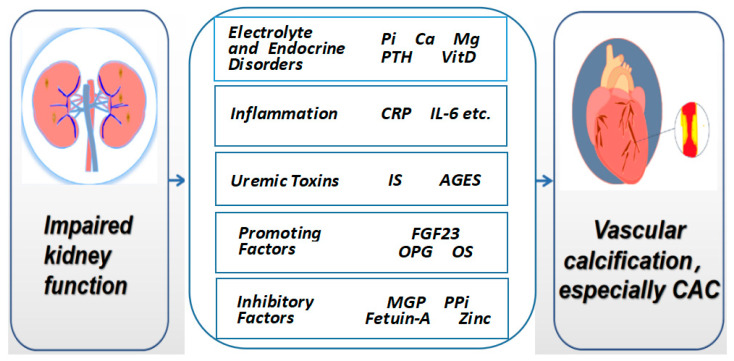
Risk factors associated with artery calcification in CKD patients. Pi: phosphorus; Ca: calcium; Mg: magnesium; PTH: parathyroid hormone; VitD: vitamin D; CRP: C-reactive protein; IL-6: interleukin-6; IS: indoxyl sulfate; AGEs: advanced glycation end products; FGF23: fibroblast growth factor-23; OPG: osteoprotegerin; OS: oxidative stress; MGP: matrix Gla protein; PPi: pyrophosphate; CAC: coronary artery calcification.

Vascular calcification is influenced by a variety of factors. Impaired renal function leads to decreased excretion and metabolic disorder. The retention of various metabolites, combined with electrolyte and endocrine disorders, inflammation and calcification promoting factors, initiates and accelerates the progression of vascular calcification (Figure 1).

### 2.2. Electrolyte Disorders

In the early stage of CKD, electrolytes can remain within a normal range due to the compensatory renal function. As the disease deteriorates, the excretion and metabolic functions of the kidneys continue to decline and an imbalance of water and various electrolytes gradually appears, represented by the disorder of phosphorus, calcium, and magnesium, which are closely related to the occurrence of vascular calcification (Table 1).

An elevated blood phosphorus level is the main cause and risk factor for CAC. This phenomenon is more prominent in patients with CKD. High phosphorus levels were associated with increased CVD and mortality in patients with renal insufficiency, whereas levels within the normal range were related to a lower incidence of CAC in people with normal renal function [22]. Wang et al. studied 77 patients with maintenance hemodialysis (MHD) and discovered that blood phosphorus variability was an independent predictor of CAC during follow-up, and maintaining stable serum phosphorus levels may lead to a lower CACS and decreased mortality [23]. A positive correlation between elevated blood phosphorus and CAC severity has been indicated in hemodialysis patients, which is more common and severe in patients with ESRD and correlated with ischemic CVD [24]. Adeney et al. found that higher serum phosphate concentrations (within the normal range) were strongly associated with a higher incidence of CAC in stage 3 CKD patients; each 1 mg/dL increase in blood phosphorus concentration was associated with a 21% increase in the incidence of CAC. It remains controversial whether lowering phosphate concentration affects the risk of calcification in patients with CKD [25].

Phosphate not only deposits in the vascular or endothelial layer, but also stimulates the expression of VSMC-related osteogenic transcriptional cytokines and promotes the osteogenic-like transition of VSMCs [26]. Wnt/β-catenin signaling activates the expression of Runt-related transcription factor 2 (Runx2), a key transcription factor that induces calcification and contributes to the phenotypic transformation of VSMCs [27,28]. By activating Wnt/β-catenin signaling, high phosphorus concentration can regulate Pit-1 at the transcriptional level [29]. In ESRD, high phosphorus concentration, persistent inflammatory state, and worsening uremic environment increase Pit-1 expression in VSMCs, which plays a key role in the calcification process [30]. High phosphate levels may also activate the TLR4/NF-κB signaling pathway and initiate the osteogenic-like phenotypic transformation program in VMSCs [31]. Further, high phosphorus levels induce methylation of the SM22α promoter, which has been shown to be an important event in VSMC calcification [32].

Calcium-salt-related effects also play a vital role in the calcification process in CKD patients. Clinical studies have suggested that hypocalcemia is linked to the progression of cardiovascular calcification and increases mortality in both dialysis and non-dialysis CKD patients [33]. Blood calcium can be combined with phosphorus to form calcium phosphate deposits in soft tissues, leading to ectopic calcification and lower blood calcium levels, which may result in renal osteodystrophy and secondary hyperparathyroidism, causing further calcification [34]. Lower blood calcium levels lead to intracellular calcium overload, induce more intravascular plaque formation, and hasten the development of CVD [35]. This may partly explain the association between lower blood calcium levels and an increased risk of cardiovascular mortality. Unlike hypocalcemia, hypercalcemia impairs VSMCs, changes the vascular tone, and increases blood pressure, which in turn induces atherosclerosis and calcification, leading to an increased cardiovascular burden [36]. Data from the Dialysis Prognosis and Practice Patterns Study (DOPPS) demonstrated a strong positive correlation between serum protein-corrected blood calcium and total mortality cardiovascular mortality rates [37].

High calcium and high phosphorus levels have synergistic effects on vascular calcification. An experiment proved that vascular calcification was markedly higher in rats exposed to high levels of calcium and phosphorus than to only high levels of phosphorus [38]. It is believed that high calcium levels mediate calcification, probably by inducing apoptosis, a process that promotes the release of stromal vesicles, together with apoptotic VSMCs, may serve as a nidus for calcium and phosphorus deposition [39]. When hypercalcemia and hyperphosphatemia co-occur in hemodialysis patients, the development of CAC and aortic calcification is hastened [40]. Therefore, serum calcium and phosphorus in CKD patients should be maintained at normal levels [41].

Some studies have pointed out that magnesium may have an inhibitory effect on vascular calcification. Magnesium binds to phosphate and inhibits the conversion of calcium and phosphorus to hydroxyapatite, passively interfering with calcium salt deposition [42]. Magnesium suppresses VSMC osteogenic differentiation by inhibiting the Wnt/β-catenin signaling pathway and activating the calcium sensing receptor in VSMCs [43,44]. Magnesium is also involved in the modulation of oxidative stress and protection of endothelial cell function, which is related to a reduced risk of CVD in patients with CKD; however, whether this directly prevents calcification remains unclear [45]. An experimental animal model study of CKD confirmed that increased dietary magnesium intake inhibits abdominal vascular calcification [46]. A study reported that serum magnesium in ESRD patients was negatively associated with CAC, and this association was more pronounced in those patients with high serum phosphorus concentrations (>1.40 mmol/L) [47]. A cross-sectional study involving 80 peritoneal dialysis patients found that a 0.1 mmol/L increase in serum magnesium is independently linked with a 1.1-point decrease in the abdominal aortic calcification score, suggesting that effective suppression of calcification probably existed with certain levels of serum magnesium [48]. Braake’s study showed that the higher the blood magnesium concentration, the lower the risk of vascular calcification in dialysis patients [49]. Therefore, appropriate supplementation of magnesium is hypothesized to be a promising option for treating vascular calcification in CKD. An interim analysis of a randomized double-blind placebo-controlled trial in patients with stage 3–4 CKD showed that magnesium oxide treatment was effective in slowing the progression of CAC; however, it did not suppress the progression of calcification in the thoracic aorta. More and larger trials are needed to confirm this finding [50]. In addition, it is worth noting that high magnesium levels can cause gastrointestinal discomfort and other adverse effects, including cardiac arrest in severe cases. It is still uncertain which range of blood magnesium concentrations is tolerable and most ideal in CKD patients.

**Table 1:** Mechanisms by which electrolyte disorders affect vascular calcification.

Electrolyte	Mechanisms	Ref.
Phosphate	Hyperphosphatemia stimulates the expression of VSMC-related osteogenic transcriptional cytokines and promotes the osteogenic-like transition of VSMCs.	[26]
	Hyperphosphatemia activates Wnt/β-catenin signaling and increases Pit-1 expression.	[29,30]
	Hyperphosphatemia activates the TLR4/NF-κB signaling.	[31]
	Hyperphosphatemia induces methylation of the SM22α promoter.	[32]
Calcium	Hypocalcemia combines with high blood phosphorus leading to ectopic calcification and secondary hyperparathyroidism.	[34]
	Hypocalcemia leads to intracellular calcium overload, mediating intravascular plaque formation.	[35]
	Hypercalcemia impairs VSMC function, causing changes in vascular tone.	[36]
	Hypercalcemia promotes apoptosis and VSMC matrix vesicle release, providing hydroxyapatite nucleation sites.	[39]
Magnesium	Hypomagnesemia inhibits the conversion of calcium and phosphorus to hydroxyapatite and passively interferes with calcium salt deposition.	[42]
	Hypomagnesemia inhibits the Wnt/β-catenin signaling pathway and activates the calcium-sensing receptor in VSMCs.	[43,44]
	Hypomagnesemia participates in the regulation of oxidative stress and protects endothelial cell function.	[45]

### 2.3. Parathyroid Hormone and Vitamin D

During the development of intermediate to advanced CKD, disturbances of phosphorus and calcium are usually associated with secondary metabolic disorders, such as secondary hyperparathyroidism and vitamin D deficiency. High parathyroid hormone (PTH) secretion influences the expression of proinflammatory cytokines and plays a passive role in the modulation of vascular remodeling [51]. High PTH levels are potentially toxic and contribute to the conversion of VSMCs to osteoblasts, causing atherosclerosis and vascular and arterial valve calcification, which increase the risk of CVD and all-cause mortality [52]. High PTH stimulates the activation of the protein kinase pathways, facilitates the expression of advanced glycation end products (AGEs) and interleukin-6 (IL-6), and indirectly promotes calcification [53]. Clinical studies have shown that high PTH levels in dialysis patients are an independent risk factor for vascular calcification [54,55]. Hartmut et al. demonstrated that nine times higher than normal PTH levels were closely associated with CAC progression in ESRD [56]. In addition, low PTH is likewise a risk factor for death in dialysis patients [57], and this category appears to be more susceptible to cardiovascular calcification [54]. The reason might be related to the downregulation of bone resorption of phosphate and calcium as a result of a low PTH-mediated reduction in the bone conversion rate.

Vitamin D is an essential steroid hormone in the body, which has a unique role in the modulation of the vascular function. It can prevent endothelial cell calcification by inhibiting cholesterol outflow and foam cell formation, regulate the renin–angiotensin system, and improve hemodynamics [58]. Vitamin D deficiency leads to impaired cardiovascular function in CKD patients [59]. In animal studies, diffuse calcification involving the aortic intima was found in uremic rats receiving relatively low doses of oral calcitriol (0.25 mg/kg/day) [60]. In patients with glomerular disease, if vitamin D is deficient, the activity of pro-inflammatory factors is enhanced, which mediates calcification [61].

However, a study indicated that the long-term use of cholecalciferol, the nutritional form of vitamin D, failed to reduce the development of vascular calcification in patients with vitamin D deficiency-induced CKD [62]. Similar results were obtained in childhood ESRD patients and found the use of active vitamin D drugs was positively correlated with CAC [63]. 

In addition, one study also found that both low and high vitamin D levels were associated with vascular calcification (U-curve relationship) in pediatric patients treated with dialysis [64]. In a cross-sectional study involving 80 CKD patients, no association was found between the degree of vitamin D deficiency and CAC [65]. 

Thus, the association between vitamin D levels and vascular calcification needs to be further explored.

### 2.4. Inflammation

Chronic inflammation significantly influences the progression of arterial calcification and atherosclerosis. In the presence of risk factors and damage, endothelial cells and macrophages degenerate, the permeability of low density lipoprotein (LDL) increases, and more LDL deposits in the intima of the vessel wall, and subsequently an inflammatory response is initiated. This process, in turn, stimulates endothelial cells and activates the BMP/Smad signaling pathway, which causes VSMC osteogenic differentiation.

Chronic inflammatory states are prevalent in CKD patients. Inflammation can increase alkaline phosphatase expression and decrease α-smooth muscle actin expression, stimulate VSMC conversion to an osteogenic phenotype, and calcify vessels in CKD patients. Mechanistically, disruption of the LDL receptor pathway induced by inflammation is closely related to the increased expression of BMP-2 and type I collagen, accelerating the progression of calcification, a process similar to the role of inflammation in the atherosclerotic process [66]. A previous study has suggested that IL-6 and CRP are risk factors for the development of vascular calcification in CKD patients [67]; their levels were significantly higher in patients with high CACS values (>400 points) than in those with low CACS values (<10 points) in peritoneal dialysis patients [68]. Both were positively correlated with CACS and the common carotid artery intima-medial thickness index in MHD patients [69]. High CRP levels accelerate the development of CAC in ESRD cases [70]. A follow-up study of uremic patients on peritoneal dialysis showed that CRP was an independent risk factor for the occurrence of CAC [71]. High CRP levels might be one of the key mediators of vascular wall calcification in uremic patients [72]. Taken together, the evidence demonstrates that inflammation and associated factors play a significant role in vascular calcification, particularly CAC, in CKD.

### 2.5. Uremic Toxins

Uremic toxins are substances that persistently accumulate in patients with CKD owing to a decreased eGFR and cause various clinical signs and symptoms. Uremic toxins can trigger adverse reactions, such as immune disorder and inflammatory damage. Studies have shown that the interaction between uremic toxins and inflammatory damage can directly increase the risk of vascular calcification in patients with ESRD [73]. Uremic toxins can be grouped into three categories: small (molecular mass < 500 Da), medium (500–5000 Da), and large (>5000 Da) molecules. Compared with small molecules, large molecules are less likely to be effectively removed using conventional dialysis, are more difficult to treat, and have greater associations with cardiovascular lesions.

AGEs are the end-outputs of a series of catalytic processes for large molecules, such as proteins, amino acids, and lipids. The kidneys play a key role in AGE metabolism [74]. AGEs have been shown to induce VSMC calcification by mediating oxidative stress, partly involving Nox1 (an NADPH oxidase) [75]. Due to decreased renal function and upregulation of oxidative stress in vivo, AGEs accumulate and elevate continuously in the plasma of both diabetic and non-diabetic patients with CKD [76]. Receptor of AGEs (RAGE) is a transmembrane cellular receptor for AGEs; the AGE–RAGE signaling pathway increases oxidative stress and activates many intracellular pathways that leads to the production of pro-inflammatory cytokines, including IL-6, TNF-α, and TGF-β [77]. This adversely affects endothelial cells and VSMC function, which are closely linked to vascular stiffness and atherosclerosis. The interaction of AGEs with RAGE also activates NF-κB and oxidative stress, leading to the expression of atherosclerosis-related genes, such as vascular cell adhesion molecule-1 (VCAM-1), intercellular adhesion molecule-1 (ICAM-1), plasminogen activator inhibitor-1 (PAI-1), and monocyte chemoattractant protein-1 (MCP-1), resulting in vascular calcification [78]. AGEs and oxidative stress are discovered to be strongly associated with extensive CAC in hemodialysis patients [79], and the continued accumulation of AGEs was found to be positively linked to CACS in peritoneal dialysis patients [80].

Indoxyl sulfate (IS) is a uremic toxin that combines with serum proteins and cannot be effectively removed by hemodialysis. IS promotes calcification by inducing oxidative stress in VSMCs and stimulates osteoblast-associated protein release [81]. It is involved in IL-6 expression in endothelial cells and VSMCs via the OAT3/AhR/NF-kB pathway [82], as well as in the secretion of IL-8 by endothelial cells under conditions with high phosphorus levels, which can promote calcification [83]. Studies have shown that the regulation of Pit-1 expression affects calcification. IS promotes VSMC calcification by partially promoting Pit-1 expression via activation of the JNK pathway [84]. It can also downregulate miR-29b activity and activate Wnt/β-catenin signaling to promote calcification [85]. Klotho is a membrane protein that is significantly expressed in kidney, parathyroid, and brain tissues. Previous studies have demonstrated that the Klotho protein has a protective effect on the kidneys of patients with CKD [86]. In rats, high levels of IS regulate the transcriptional process of vascular Klotho and reduce the expression of Klotho. The epigenetic modification of Klotho by IS may contribute to vascular calcification at the end-stage of CKD [87]. Barreto’s data showed that elevated IS was correlated with a higher occurrence of aortic calcification in patients with CKD [88] and paralleled the severity of calcified vessels in ESRD patients [89].

**Figure 2:**
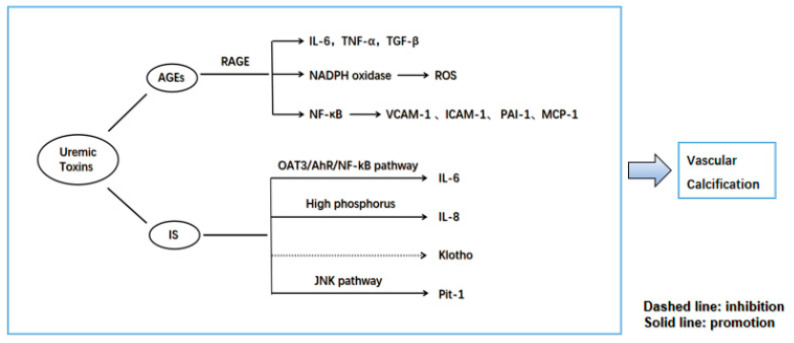
Involved factors associated with AGEs and IS to promote vascular calcification. AGEs: advanced glycation end products; RAGE: receptor for AGEs; IL-6: interleukin-6; TNF-α: tumor necrosis factor alpha; TGF-β: transforming growth factor beta; ROS: reactive oxygen species; NF-κB: nuclear factor-κB; VCAM-1: vascular cell adhesion molecule-1; ICAM-1: intercellular adhesion molecule-1; PAI-1: plasminogen activator inhibitor-1; MCP-1: monocyte chemoattractant protein-1; IS: indoxyl sulfate.

The possible pathways by which AGEs and IS mediate vascular calcification are shown in Figure 2. Different uremic toxins have different mechanisms and principles of action. Current studies have provided some evidence of a relationship between uremic toxins and calcification, most of which are still focused on small-molecule toxins. It is expected that additional unknown uremic toxins will be identified to reveal the pathogenesis of cardiovascular calcification in CKD.

### 2.6. Fibroblast Growth Factor 23

Fibroblast growth factor-23 (FGF23) is a cytokine synthesized, secreted, and released primarily by osteoblasts and osteoclasts [90]. It stimulates phosphorus excretion by the kidneys and reduces phosphorus absorption from the diet by inhibiting the synthesis of 25(OH)D3 [91,92]. FGF23 is mainly metabolized by the kidneys, and its clearance reduces as the renal function decreased. FGF23 may cause endothelial dysfunction by directly disrupting nitric-oxide-mediated vasodilation [93]. Clinical investigations showed that an increase in FGF23 levels is positively correlated with aortic calcification and CAC in patients with CKD at different stages [94,95,96]. High FGF23 levels may be a new factor contributing to ectopic calcification in CKD [97].

FGF23 can bind to Klotho, which is required for the activation of FGF23 and its receptors, and further increase their affinity. The Klotho–FGF23 axis signaling pathway is closely related to the regulation of calcium and phosphorus metabolism, PTH homeostasis, and CKD-related complications [98]. α-Klotho can be used as a humoral agent to promote renal phosphorus excretion. Klotho in patients with CKD continues to decrease with declining renal function from the early stages of CKD [99], and its deficiency accelerates calcification [100]. Klotho suppresses the progression of vascular calcification by several mechanisms: (1) acts on sodium–phosphorus co-transport proteins (Pit-1 and Pit-2) to downregulate phosphorus uptake by VSMCs and inhibit Runx2 protein expression [5]; (2) inhibits oxidative stress and attenuates oxidative damage and apoptosis [101]; (3) prevents vascular calcification by partially inhibiting the Wnt/β-catenin signaling pathway [102]; (4) directly suppresses phosphorus-induced calcification and hinders the conversion of VSMCs to osteoblasts [103].

There were also different results for FGF23. A correlation between blood FGF23 levels and CAC was not found in more than 1000 patients with stages 2–4 CKD [104]. Lau et al. did not detect α-Klotho mRNA in the aorta of mice in the normal and CKD groups [105].

It is still questionable whether FGF23 can be used as an indicator of CAC; further research is also required to identify whether the anticalcification effects of Klotho act directly or indirectly through alterations in serum phosphate or other agents.

### 2.7. Osteoprotegerin

Osteoprotegerin (OPG) is a soluble glycoprotein that is widely expressed in various tissues and cells. It is an osteoclast inhibitory factor. Receptor activator of nuclear factor-κB ligand (RANKL) proteins are thought to be essential biomolecules in the process of osteoclast activation and proliferation and can interact with RANK protein to promote bone differentiation and resorption. RANKL promotes vascular calcification by upregulating BMP4 expression to activate Wnt signaling and mediates BMP2 release from vascular endothelial cells [106,107]. OPG, which is structurally similar to RANK, competitively interacts with RANKL, leading to the inhibition of osteoclast differentiation from osteoclast precursor cells and bone resorption, with reductions in bone loss [108,109]. RANKL binding to RANK mediates VSMC osteogenic phenotypic differentiation, expression and release of bone matrix proteins, and promotes calcification, whereas OPG can counteract the calcifying effects by neutralizing RANKL [110]. OPG may serve as a bridge between bone metabolism and vascular diseases.

OPG knockdown mice show significant aortic and renal artery calcification rates [111]. In contrast to its protective effect in animal models, OPG appears to correlate with the severity of calcification in human studies. Serum OPG concentration was positively correlated with serum creatinine level and significantly higher in dialysis patients than in the healthy population [112]. Its level was correlated with the degree of aortic calcification in patients undergoing hemodialysis [113]. It is an independent predictor of the calcification score, and high serum concentration favored the development of CAC [114,115]. Mesquita et al. showed that OPG is an independent risk factor for death in patients with CKD and an early predictor of CAC [116]. A long-term follow-up study of 47 patients with MHD found that patients initially free of vascular calcification remained free of calcification at the end of the study, and the levels of OPG in these patients were consistently and significantly lower than in patients with calcification. Additionally, significant higher levels of OPG and more severe vascular calcification were found in dead patients [117]. OPG may serve as an important predictor of atherosclerosis and vascular calcification in patients with ESRD. Marques et al. reported that high serum OPG levels are strongly associated with the occurrence of adverse cardiovascular events in patients with CKD [118]. A prospective study of renal transplant patients also showed that serum OPG was an independent predictor of cardiovascular death [119]. Although studies have shown that OPG is associated with the development of vascular calcification and the occurrence of adverse cardiovascular events, the exact mechanism remains unclear. Further studies are required to confirm the specific role of OPG.

### 2.8. Matrix Gla Protein

Matrix Gla protein (MGP) belongs to the Gla protein family and is mainly secreted by VSMCs in the arterial walls. MGP has been shown to be effective in inhibiting vascular calcification both in vitro and in vivo [120]. MGP requires γ-carboxyglutamylation and phosphorylation to be activated and exert its effects. Vitamin K is a key enzyme involved in carboxylation. In a mouse model, the administration of vitamin K antagonists for several weeks resulted in insufficient carboxylation of MGP in vivo, which accelerated the development of aortic calcification. This indicated that MGP requires vitamin K to trigger biological activity [121]. BMP-2 converts VSMCs to the osteoblast phenotype. Only through carboxylation and phosphorylation can MGP gain the capacity to combine calcium and BMP-2, thereby suppressing calcification [122]. Early subclinical microcalcifications in the coronary arteries are often produced if MGP carboxylation is insufficient [123]. Additionally, MGP could also inhibit calcification by binding Ca^2+^ and hydroxyapatite crystals and forming fetuin-A–MGP-mineralization complexes [124,125].

A poor vitamin K status in the CKD setting has been repeatedly confirmed [126]. In animal models, exogenous vitamin K supplementation showed promising results. Rats with renal failure treated with vitamin K1 and K2 for 4 weeks showed a significant reduction in renal and aortic calcification [127]. McCabe et al. treated CKD mice with high dietary vitamin K1 was ultimately found to be effective in slowing the progression of arterial calcification [128]. However, studies in populations have shown different results. In a cohort of 42 patients with CKD stages 3–5, daily intake of 90 µg vitamin K2 resulted in slower CAC progression [129]. In contrast, several other studies have shown that vitamin K supplementation did not improve cardiovascular calcification in patients with CKD or dialysis [130,131,132]. Therefore, further large datasets are needed to support the efficacy of vitamin K treatment in these patients to delay the progression of vascular calcification.

A study in 97 ESRD patients found no correlation between MGP and vascular sclerosis and CACS [133]. Mizuir et al. also showed that total MGP levels in MHD patients were substantially higher than those in the control groups, but were not related to CACS [134]; this may be related to the fact that the carboxyl and non-carboxyl forms of MGP were not distinguished in this study. However, another study showed that a high expression of MGP in vascular tissue was linked to a higher CACS and plasma dp-ucMGP (dephosphorylated non-carboxylated MGP) levels in CKD stage 5 patients. Additionally, a prospective study of more than 100 patients at different stages of CKD found that dp-ucMGP was strongly associated with aortic calcification scores and all-cause mortality [135,136]. To summarize, larger controlled trials ought to test the effect of vitamin K intake (via carboxylation of dp-ucMGP) on vascular calcification and CVD in patients with CKD.

### 2.9. Fetuin-A

Fetuin-A is a negatively charged binding glycoprotein that is synthesized by the liver and released into the bloodstream. It has been proven to be an effective systemic inhibitor of calcification [137]. It inhibited VSMC calcification induced by elevated extracellular mineral ion concentrations, which was mainly achieved by inhibiting apoptosis and cysteine protease cleavage [138]. It may inhibit vascular calcification by blocking the BMP signal transduction pathway, downregulating the release of inflammatory factors and inhibiting inflammatory activity [139,140,141].

A multicenter cohort study found that an increased risk of cardiovascular death was associated with decreased expression of serum fetuin-A after seven years of follow-up in approximately 1000 CKD dialysis patients [142]. The fetuin-A levels decreased as CKD progresses, and its deficiency was associated with an increased tendency for systemic calcification and a poorer prognosis [143]. In MHD patients, lower fetuin-A levels were associated with a more severe total CACS [144], and the CACS, mass, and volume of coronary plaques were significantly correlated with fetuin-A levels [145]. However, the fetuin-A levels are significantly higher in diabetic nephropathy patients who have not yet undergone maintenance dialysis than in diabetic control patients, and are directly associated with CAC. The reason for this contradictory result may be that fetuin-A upregulation is probably a defense mechanism against early vascular calcification [146]. Fetuin-A plays a key role in the combination and clearance of calcium-mineralized substrates accumulated in the fetal hypoxic kidneys and acts as an inhibitor of ectopic calcification, maintaining the integrity of the kidney tissue and averting the advancement of CKD [147]. However, other studies have reported different results. A study showed that there was no correlation between fetuin-A levels and vascular calcification in patients with MHD [148]; a correlation was also not observed between fetuin-A levels and CACS in 85 patients with diabetic nephropathy [149]. In another study, vascular calcification was not affected by the fetuin-A and bone-bridging protein levels in hemodialysis patients [150]. Whether fetuin-A can inhibit vascular calcification, especially CAC, in each stage of CKD, remains to be confirmed in future prospective studies.

### 2.10. Pyrophosphate

Pyrophosphate (PPi) is synthesized and released by VSMCs, and its catabolism is mainly performed by tissue-non-specific alkaline phosphatase (TNAP). TNAP is a key determinant of tissue pyrophosphate levels. It controls vascular calcification by affecting the synthesis and hydrolysis of extracellular pyrophosphate (ePPi) [151]. ePPi may be a major inhibitor of vascular calcification, and a lack of extracellular enzymes for ePPi synthesis induced the calcification of large portions of the rat aorta [152]. Daily peritoneal dialysis with a solution containing PPi significantly inhibited calcification progression in a CKD mouse model [153]. In patients with CKD and ESRD, decreased concentrations of PPi are closely correlated with the development of arterial calcification [154,155]. The supplementation of exogenous calcifying agents such as PPi during dialysis prevents dialysis-related calcification in patients [156]. Targeted methods that interfere with pyrophosphate metabolic processes to increase pyrophosphate levels in vivo and inhibit the onset of calcification are expected to be effective in the treatment of vascular calcification.

### 2.11. Zinc

Zinc is an essential trace element for maintaining the normal structure and function of human cells, mainly stored in the kidneys and liver, and involved in the synthesis of many coenzymes in the body. In experimental models, the effects of zinc on the vasculature have been described in detail, with zinc deficiency exacerbating intimal atherosclerosis [157]. In patients on long-term hemodialysis, decreased serum zinc levels were also associated with an increased carotid intima and mesothelial thickness [158]. Zinc deprivation inhibits extracellular matrix calcification in osteoblasts by inhibiting the accumulation of calcium and phosphorus and reducing the synthesis and activity of type I collagen, matrix proteins, and alkaline phosphatase [159]. Voelkl et al. demonstrated that zinc sulfate upregulated tumor necrosis factor-α-induced protein 3 (TNFAIP3) gene expression in a high phosphorus environment through zinc-sensitive receptor and elevated TNFAIP3 levels inhibited the activation of NF-κB, a transcription factor and key regulator of the inflammatory response pathway, thereby inhibiting the transdifferentiation of contractile VSMCs to the bone/chondrocyte phenotype and reducing calcification [27]. Nagy et al. [160] showed that hypoxia-inducible factor (HIF) promotes the loss of VSMC markers and the expression of osteogenic genes, and exacerbates phosphorus-induced osteogenic differentiation in VSMCs, while zinc inhibits this process in a dose-dependent manner.

Several observational studies had shown that low dietary zinc intake is associated with cardiovascular disease mortality [161,162], and the serum zinc levels were found to be lower in patients with MHD than in the healthy population [163]. Recently, Chen et al. found that high dietary zinc intake was independently associated with a lower risk of severe abdominal aortic calcification in more than 2000 US non-institutionalized adults. In that study, 18.1% of the subjects were CKD patients [164]. In addition, some studies have reported that diets with a given magnesium/zinc ratio are associated with an increased risk of CAC progression, which is mediated by pro-calcification IL-6 [165]. 

In conclusion, correction of hypozincemia may be a simple and effective clinical approach to reduce the progression of vascular calcification and cardiovascular disease in patients with CKD. This deserves further investigation.

### 2.12. Oxidative Stress

Oxidative stress is the generation of reactive oxygen (ROS) molecules during cellular respiration in the organism under pathological conditions that exceed the scavenging capacity of the organism, causing damage to the organism by reactive free radicals. Oxidative stress, which is involved in vascular calcification, is present in CKD in various forms, but ROS are considered to be the main mediators mediating vascular calcification [166], cardiovascular events [167] and other complications. The main ROS molecules are superoxide anion (O_2_^−^), hydrogen peroxide (H_2_O_2_), and hydroxyl radicals. Oxidative stress may be involved in vascular calcification in CKD through the induction of osteogenic degeneration of VSMCs [168,169,170], alteration of calcification regulators [171], and imbalance of antioxidant systems.

The nuclear factor erythroid-2-related factor 2 (Nrf-2), a protein encoded by cDNA, is one of the important transcription factors regulating the adaptive antioxidant response of the organism [172]. Nrf-2 is involved in the regulation of heme oxygenase (HO-1), NADPH quinine oxidoreductase (NQO-1) and glutathione reductase, which plays a protective role against oxidative stress [173]. Under physiological conditions, Nrf-2 is stored in the cytoplasm attached to Keap-1, degraded by the proteasome after ubiquitination; when subjected to oxidative stress, Nrf-2 dissociates from Keap-1 and binds to antioxidant response elements through small Maf to regulate the expression of downstream genes [174]. Keap1-Nrf2 plays an important role in maintaining intracellular metabolism and adapting to oxidative stress. It has been found that the inhibition of Nrf-2 and downstream antioxidant protein HO-1 activity exacerbated inflammation and oxidative-stress-induced renal injury in a rat model of adenine-diet-induced chronic renal interstitial tubular disease [175]. Recently, it was found AGE-modified bovine serum albumin induces ROS production in bovine aortic endothelial cells and activates two Nrf-2-dependent antioxidant genes, HO-1 and NQO1, to counteract ROS-induced damage [176]. Liu et al. observed that leucovorin protected human keratinocytes from oxidative stress induced by UV radiation and enhanced the activity of antioxidant enzymes [177]. A recent study has shown that upregulation of the Nrf2 system can attenuate the high Pi-induced calcification levels in VSMCs [178].

More clinical and experimental studies are needed in the future to further investigate the specific mechanisms of oxidative stress in promoting vascular calcification formation in CKD patients and find effective therapeutic measures.

Clinical studies about the factors associated with CAC are shown in Table 2.

## 3. Research Progresses

Except mentioned above, many new drugs may be effective in inhibiting the progression of CKD calcification and have a protective effect on the cardiovascular system. Phosphorus-binding agents can control hyperphosphatemia and are currently the mainstay of prevention and treatment of abnormalities in mineral and bone metabolism in patients with CKD. Results from a study of patients with stage 3–4 CKD showed that their CAC progression was significantly reduced by combining a phosphorus-restricted diet with sevelamer [179]. After treatment with the new phosphate-binding agent calcium–magnesium tablets for months, hemodialysis patients showed significantly lower levels of aortic valve calcification [180]. The types of phosphorus-binding agents currently on the market and under development include calcium-containing phosphate-binding agents, lanthanum carbonate, and iron-containing phosphate-binding agents, etc.

Bisphosphonates, as pyrophosphate analogues, could be taken up by osteoclasts and inhibit the enzymes necessary for bone resorption, effectively inhibiting calcium hydroxyapatite formation [181]. In a prospective trial in renal transplant patients, alendronate was found to be effective in treating secondary osteoporosis and inhibiting the progression of abdominal aortic calcification [182]. The RANKL inhibitor denosumab effectively elevates OPG protein expression and inhibits RANKL protein expression, resulting in increased calcium and phosphorus deposition in bone tissue and decreased deposition in blood vessels, providing relief for both vascular calcification and osteoporosis; effectively inhibits the progression of CAC in dialysis patients and abrogates bone calcification in severe cases with high bone turnover rates [183]. The long-term use of denosumab has been shown to reverse or treat the calcification of the aortic arch in hemodialysis patients [184].

Calcimmimetics can improve the calcium sensitivity of parathyroid calcium-sensitive receptors and reduce calcium, phosphorus, and PTH levels [185], not only to control secondary hyperparathyroidism in patients with CKD, but also to improve vascular calcification and maintain normal bone metabolism. An RCT study showed that vascular and cardiac valve calcification progressed more slowly in patients in the cenacalcid plus low-dose vitamin D group [186]. A new intravenous preparation, etelcalcitide, has been reported to lower PTH more potently and for a longer duration than cenacalcid, but is prone to hypocalcemia and prolonged ECG QT intervals [187]. Therefore, the optimal timing of calcium mimetics, when to discontinue, the duration of treatment, and long-term safety issues still need to be studied in depth.

Phytate is an endogenous crystallization inhibitor that is closely associated with calcification-related diseases. Small molecular weight and water solubility make it dialyzable and its loss exacerbates the development of calcification in dialysis patients [188]. An intravenous formulation of hexabenzodicarboxylate (SNF472) has been developed to address this phenomenon and slow the progression of calcification and CAC in CKD patients. The results of a large double-blind, placebo-controlled RCT showed that SNF472 delayed the progression of CAC and aortic valve calcification in hemodialysis patients compared to a placebo [189].

In addition, many modulators of mineral handling and calcification in CKD, such as odanacatif (anticathepsin K), romosozumab (antisclerostin antibody), and SGLT (sodium glucose convertor)-2 inhibitors are under investigation. 

## 4. Conclusions and Perspective

A high incidence of systemic vascular calcification, especially CAC, is a major risk factor of CVDs in patients with CKD. However, there are no clearly proven methods to effectively reverse it. More researched to further explore and elucidate the relationship between CKD and vascular calcification may be helpful to provide effective therapeutic targets and reduce the occurrence of vascular events. With the in-depth exploration of the mechanism and the advancement of various drug development and clinical trials, the future of CKD calcification treatment remains worthy of anticipation.

## Figures and Tables

**Table 2:** Clinical studies about the factors associated with CAC.

Factors	Subjects	Conclusion	Whether Related to CAC	Ref.
Phosphorus	77 HD patients	Blood phosphorus variability was an independent predictor of CAC, and maintaining stable serum phosphorus levels may lead to a lower CACS.	Yes	[23]
	205 HD patients	A positive correlation existed between elevated blood phosphorus and CAC severity, and was associated with ischemic CVD.	Yes	[24]
	439 CKD patients in stage CKD 3	Higher serum phosphate concentrations (within the normal range) were strongly linked with a high incidence of CAC; each 1 mg/dL increase in blood phosphorus concentration was linked with a 21% increase in the incidence of CAC.	Yes	[25]
Calcium, phosphorus	200 HD patients	When hypercalcemia and hyperphosphatemia co-occur, the development of CAC and aortic calcification is hastened.	Yes	[40]
Magnesium	109 CKD patients	Serum magnesium in ESRD patients was negatively associated with CAC; this association was more pronounced in patients with high serum phosphorus concentrations.	Yes	[47]
	324 CKD patients in stage CKD 3–4	Magnesium oxide treatment was effective in slowing the progression of CAC, but it did not suppress the progression of calcification in the thoracic aorta.	Yes	[50]
PTH	213 patients in stage CKD-5D	Nine times higher than normal PTH levels were closely associated with CAC progression.	Yes	[56]
Vitamin D	40 CKD patients	Active vitamin D drug use was positively associated with CAC.	Yes	[63]
	80 CKD patients	No association was found between the degree of vitamin D deficiency and CAC.	No	[65]
Inflammation	43 PD patients	IL-6 and CRP levels were significantly higher in patients with a high CACS (>400 points) than in those with low CACS (<10 points) values.	Yes	[68]
	73 PD patients	The CACS and the Common Carotid Artery Intima-Medial Thickness Index were positively correlated with CRP and IL-6.	Yes	[69]
	40 HD patients	High CRP levels accelerated the development of CAC.	Yes	[70]
	70 PD patients	CRP was an independent risk factor for the occurrence of CAC.	Yes	[71]
AGEs	40 HD patients	AGEs and oxidative stress were strongly associated with extensive CAC.	Yes	[79]
	27 PD patients	Continued accumulation of AGEs was found to be positively linked to CACS.	Yes	[80]
FGF23	16 HD patients	FGF23 levels were independently linked to aortic, peripheral calcification and CAC.	Yes	[94]
	142 CKD patients	Patients with elevated FGF23 levels had higher aortic and CACS values than patients with lower FGF23 levels.	Yes	[95]
	1501 patients in CKD stage 2–4	A correlation between blood FGF23 levels and CAC was not found.	No	[104]
OPG	185 CKD patients	A high serum OPG concentration favored a high CAC.	Yes	[114]
	101 HD patients	A higher OPG level was independently associated with CAC.	Yes	[115]
	77 CKD patients	OPG is an independent risk factor for death in patients with CKD and an early predictor of CAC.	Yes	[116]
MGP	97 CKD patients	MGP did not correlate with vascular sclerosis and CACS.	No	[133]
	112 HD patients	The MGP levels were substantially higher than those in the control groups, but were not related to CACS.	No	[134]
	141 patients in CKD stage 5	High vascular expression of MGP was associated with higher CAC scores and plasma dp-ucMGP levels.	Yes	[135]
Fetuin-A	78 HD patients	Serum fetuin-A levels were associated with the total CACS.	Yes	[144]
	72 HD patients	The CACS, mass, and volume of plaques in coronary arteries correlated significantly with the serum fetuin-A levels.	Yes	[145]
	88 diabetic nephropathy patients	The fetuin-A levels were significantly higher in diabetic nephropathy patients who had not yet undergone maintenance dialysis than in diabetic control patients, and were directly associated with CAC.	Yes	[146]
	85 diabetic pre-dialysis patients	There was no association between fetuin-A and CACS.	No	[149]

HD: hemodialysis; PD: peritoneal dialysis; PTH: parathyroid hormone; AGEs: advanced glycation end products; FGF23: fibroblast growth factor-23; OPG: osteoprotegerin; MGP: matrix Gla protein; CAC: coronary artery calcification; CACS: coronary artery calcification score; ESRD: end-stage renal disease; CVD: cardiovascular disease; dp-ucMGP: dephosphorylated non-carboxylated MGP.
